# *Daphnia japonica* sp. nov. (Crustacea: Cladocera) an eastern Palearctic montane species with mitochondrial discordance

**DOI:** 10.7717/peerj.14113

**Published:** 2022-10-04

**Authors:** Alexey A. Kotov, Derek J. Taylor

**Affiliations:** 1 Laboratory of Aquatic Ecology and Invasions, A.N. Severtsov Institute of Ecology and Evolution of Russian Academy of Sciences, Moscow, Russia; 2 Biological Sciences, State University of New York at Buffalo, Buffalo, NY, United States

**Keywords:** Daphnia, Zooplankton, Branchiopoda, Cladocerans, Daphniids, Endemism, Hybridization

## Abstract

The *Daphnia longispina* complex (Crustacea: Cladocera) contains several keystone freshwater species such as *D. longispina* O.F. Müller (*D. rosea* Sars is a junior synonym), *D. galeata* Sars, *D. cucullata* Sars, and *D. dentifera* Forbes. The complex is common throughout the Holarctic, but there are several geographic regions where local forms have been assigned to European species names based on a superficial morphological resemblance. Here we examine the species status of a form that was previously assigned to *D. rosea* from a montane bog pond on Honshu, Japan. We used two nuclear non-coding loci (nDNA), mitochondrial sequences (the ND2 protein-coding region) and morphology for evidence. The mitochondrial gene evidence supported the existence of a divergent lineage that is more closely related to *D. galeata* than to *D. dentifera*. However, morphology and the nuclear DNA data indicated a lineage that is most closely related to *D. dentifera*. As our evidence supported the existence of a cohesive divergent lineage, we described a new species, *Daphnia japonica* sp. nov. Recognition of local and subalpine diversity in this group is critical as ongoing anthropogenic disturbance has been associated with introductions, local extirpations, and hybridization.

## Introduction

Crustaceans of the genus *Daphnia* O.F. Müller (Crustacea: Cladocera) are model organisms in aquatic toxicology, ecology, and evolutionary genetics (Benzie, 2005; Smirnov, 2017). Although *Daphnia* is the best studied cladoceran genus, there is still taxonomic confusion. Presently, *Daphnia* is composed of three subgenera *D. (Australodaphnia)* Colbourne, Wilson & Hebert; *D. (Ctenodaphnia)* Dybowski & Grochowski and *Daphnia* s. str. DNA sequence evidence (nuclear and mitochondrial) supported these divergent subgenera (Adamowicz et al., 2009; Colbourne, Wilson & Hebert, 2006; Kotov, 2016; Omilian & Taylor, 2001). The subgenus *Daphnia* is further subdivided into two species groups: *D. pulex* and *D. longispina*. The *Daphnia longispina* group is common in Holarctic standing waters, but rare in tropical and subtropical lowlands (Dumont, 1994). The complex consists of four species complexes: *D. longiremis*, *D. laevis*, *D. curvirostris* and *D. longispina* (Adamowicz et al., 2009; Taylor, Finston & Hebert, 1998). Of these groups, the *D. longispina* species complex has perhaps the most notoriously confused taxonomy despite containing keystone freshwater species such as *D. longispina* O.F. Müller (*D. rosea* Sars is a junior synonym), *D. galeata* Sars, *D. cucullata* Sars, and *D. dentifera* Forbes. A number of factors has complicated the taxonomy: hybridization, introgression, phenotypic plasticity, and introductions (Petrusek et al., 2008; Taylor & Hebert, 1994; Thielsch et al., 2017; Wolf & Mort, 1986; Zuykova et al., 2018b). As with many cladoceran taxa, there has also been a historical tendency for regional forms to be assigned to morphologically or ecologically similar European taxa (Brooks, 1957).

In addition, the taxonomy of the *Daphnia longispina* complex has been complicated by a modest understanding of morphological evolution in the group. The group was the subject of seminal studies of phenotypic plasticity (Jacobs, 1961; Karpowicz et al., 2020; Korinek & Machacek, 1980; Lampert, 2011; Woltereck, 1919). Later, several studies focused on the size, general shape and male character evolution (Fryer, 1991; Giessler, Mader & Schwenk, 1999; Glagolev, 1984; Zuykova et al., 2018a). Initially, some structures, such as the “neckteeth” of juvenile females and males, were thought to be diagnostic of a species (Brooks, 1953). Then, neckteeth were regarded as independently derived in several species (Kotov, Ishida & Taylor, 2006). However, neckteeth could also be an ancestral trait of the subgenus *Daphnia* (Juračka, Laforsch & Petrusek, 2011; Kirdyasheva & Kotov, 2013; Sperfeld et al., 2020). Another factor adding to taxonomic confusion is paedomorphosis during the evolution of the *D. longispina* species complex (Kirdyasheva & Kotov, 2019). The males of this group often resemble the juvenile males of other groups of *Daphnia*, leading to a dearth of derived morphological characters.

A lack of knowledge of evolution can also affect the taxonomic use of DNA-based markers. Mitochondrial rRNA gene studies revealed several local divergent lineages within the *Daphnia longispina* group (Ishida et al., 2011; Petrusek et al., 2008; Petrusek, Thielsch & Schwenk, 2012). While some divergent mitochondrial lineages (*i.e*., potentially novel species) have agreed with nuclear and morphological evidence (such as *Daphnia umbra*), several divergent 12S rRNA gene sequence lineages (*i.e*., greater than 10% K2 divergence from named sister species) have lacked support from nuclear DNA markers and morphological evidence. Thielsch et al. (2017) found that three divergent 12S rDNA lineages of the *Daphnia longispina* complex lacked nuclear genetic divergence from three common species. They proposed that ancient mitochondrial introgression may have contributed to the unusual divergence with 12S rRNA genes compared to nuclear markers and morphology. PCR amplification of nuclear copies of mitochondrial genes may also contribute to mitonuclear discordances. Assays for pseudogenes (intact ORF’s and tests of purifying selection) are more straightforward for protein coding genes (compared to 12S rRNA gene sequences). Beninde (2021) provided genome scale evidence that the lineages found by Ishida et al. (2011) were in fact unique for nuclear and mitochondrial DNA (albeit discordant).

Here, we assess the species hypothesis for a montane form that we detected in Misumi-ike, Japan, which has yet to be examined at a genetic level. We use nuclear DNA sequences, morphology, and protein-coding mitochondrial gene sequences and link this form with additional montane populations and formally describe a new endemic species, *Daphnia japonica* sp. nov.

## Materials and Methods

The electronic version of this article in Portable Document Format (PDF) will represent a published work according to the International Commission on Zoological Nomenclature (ICZN), and hence the new names contained in the electronic version are effectively published under that Code from the electronic edition alone. This published work and the nomenclatural acts it contains have been registered in ZooBank, the online registration system for the ICZN. The ZooBank LSIDs (Life Science Identifiers) can be resolved and the associated information viewed through any standard web browser by appending the LSID to the prefix http://zoobank.org/. The LSID for this publication is: urn:lsid:zoobank.org:pub:7AAA1961-81B8-46D8-8D0A-9FE1F5CD776D. The online version of this work is archived and available from the following digital repositories: PeerJ, PubMed Central SCIE and CLOCKSS.

### Ethics statement

Plankton samples from Japan were collected by Dr. S. Ishida during his Ph.D. dissertation work, such sampling does not require special permission in Japan. The species was not assessed as endangered at the time of collection and is currently not subject to specific regulations, however all efforts were taken to ensure that the collection and preservation of animals was performed with due consideration of their welfare. The number of individuals taken did not represent a significant proportion of the clonal populations present at each site.

### DNA sequencing

Ethanol-preserved specimens of the *Daphnia longispina* group (see Table 1 and Table S1 in supplement, which includes sequences from Genbank and those from this study) were exposed to DNA extraction (Quickextract DNA extraction from Epicentre as modified by Ishida et al. 2007), PCR (followed Ishida & Taylor, 2007a but with 50 °C PCR Annealing temperature), and bidirectional Sanger sequencing (TACGEN). Sequences were assembled and compared to representative sequences of named Holarctic species (*e.g*., *Daphnia dentifera*, *Daphnia galeata*, and *Daphnia tanakai*) and one ND2 sequence from an unnamed lineage (Petrusek, Thielsch & Schwenk, 2012) in the *Daphnia longispina* complex using Geneious (https://www.geneious.com). Sequences (and their genomic locations) were identified from the reference genome of *Daphnia galeata* (Nickel et al., 2021) by BLASTn and included for each gene tree. A region from the ND2 gene of the mitochondrial genome was amplified and sequenced using the primers and protocol of Ishida & Taylor (2007a). As part of a larger effort to identify informative, single copy nuclear regions, we pooled genomic DNA of 500 individuals of *Daphnia dentifera* (4 mg dry mass). This total DNA was extracted using Nucleospin gDNA Cleanup (Macherey-Nagel) and exposed to restriction enzyme digestion with *Rsa* I (New England Biolabs). Purified DNA was size selected (1–3 kbp) using a 2% agarose gel, and further purified using Nucleospin gel Cleanup. After dephosphorylation using Shrimp Alkaline Phosphatase, we performed cloning with a Zero Blunt TOPO PCR cloning kit (Invitrogen, Waltham, MA, USA). Colonies were then sequenced and primers designed. Of these, two nuclear regions were successfully amplified and sequenced from the target specimens of Misumi-ike. We used the following PCR primers (custom oligos from Integrated DNA Technologies):

**Table 1:** Sampling locations for nuclear sequences of the *Daphnia longispina* complex (including *Daphnia japonica* sp. nov. from this study).

	Location	Latitude	Longitude	Taxa
Dargin Lake	Poland	54.12	21.73	*Daphnia cucullata*
Reference	Finland			*D. cucullata*
Kurobe	Kurobe 4th Dam Reservoir	36.53	137.65	*D. dentifera*
Miyagi	Miyagi-nagamuma Pond, Miyagi	38.26	140.87	*D. dentifera*
Niseko	Niseko-onuma Pond, Hokkaido	42.90	140.62	*D. dentifera*
Tochigi	Lake Sainoko, Tochigi	36.74	139.41	*D. dentifera*
Umagami	Umagami Pond, Yamagata	38.34	140.15	*D. dentifera*
Ashinoko	Lake Ashinoko	35.21	139.01	*D. galeata*
Chiba	Pond in Chiba	35.60	140.10	*D. galeata*
Fukuoka	Lake near Fukuoka Japan			*D. galeata*
Ibaragi	Kouzogawa Reservoir, Ibaragi	36.42	140.40	*D. galeata*
Nara	Pond (Yamaguchi Shrine) Nara	34.52	135.69	*D. galeata*
Gálggojávri	Glacial Lake, Norway	69.12	20.76	*D. galeata*
Reference	Sweden			*D. galeata*
Hourai	Hourai-numa Pond, Iwate	40.61	140.94	*D. japonica*
Imori	Lake Imori-ike, Niigata	36.63	138.54	*D. japonica*
Misumi-ike	Misumi-Ike	38.37	139.82	*D. japonica*
Reference	Sweden			*D. longispina* (formerly *D. hyalina*)
Midori	Lake Midori-ga-ike, Hida Mountain Range	36.58	137.60	*D. tanakai*
Mt. Zao	Pond, Mt Zao Japan			*D. tanakai*
Akan	Akan Ko	43.43	144.09	*Daphnia galeata*
Akita	A pond in Nikaho-Kogen, Akita	39.22	140.00	Putative *D. galeata* hybrid
Aomori	Ichiyanagi Numa Pond, Rokkasho village, Aomori Prefecture	40.91	141.36	Putative *D. galeata* hybrid
Koke, K-d1, K-d2, K-g	Hybrid clones, Japan			Putative *D. galeata* hybrid
Toyama	A small man-made pond for irrigation near Toyama City	36.71	137.21	Putative *D. galeata* hybrid

**Note:**

GenBank accession numbers for the nuclear loci from this study are consecutive entries in the following range: OL412563–OL412665.

Locus 1 (F: TTTACCGATGGGCCGACCAGATTAGAG, R: GCATCCACTTGTCAGCGCCGTTTGTCA),

Locus 2 (F: CCTGTTAAAATCAACAATAACAAATAGGAA, R: GCCAATTTTATACGATTTGATGTTATGC). A nucleotide BLAST to the reference *Daphnia galeata* genome indicated that these loci are present as intergenic single copies and located on contig CAKKLH010000168 from bases 1032297 to 1032629 (locus 1) and contig CAKKLH010000342 from bases 156848 to 157308 (locus 2). Cloning (with a Zero Blunt TOPO PCR cloning kit, Invitrogen) was used for sequences from putative hybrids.

The ND2 region was aligned by translation alignment in Seaview (Gouy, Guindon & Gascuel, 2010) using the invertebrate mitochondrial genetic code. The nuclear loci (nDNA) were aligned using MAFFT (Katoh & Standley, 2014). Codon-specific partitioned models were estimated in IQtree (Nguyen et al., 2014) for the ND2 alignment. Standard model-fitting was applied to the nDNA alignment. The best fit substitution models were TN+F+G4 for nuclear locus one, and HKY+F+G4 for locus 2. Best fit substitutions models for the partitioned codon alignment of the ND2 gene were HKY+F+I, TPM3+F+G4, and TN+F. The best fit model for the amino acid alignment of ND2 was mtVer+G4. Tests of branch-specific relaxed purifying selection were made using the RELAX routine of HyPhy (Wertheim et al., 2015). Maximum Likelihood estimates of phylogenies were made in IQtree with optimal models. Two support values were estimated: approximate likelihood ratio tests and ultrafast bootstraps. Average K2+G distances among clades were estimated in Geneious (https://www.geneious.com) with the species identification plugin.

### Plankton sampling and morphological analyses

As specimens from Misumi-ike have been associated with *Daphnia dentifera* (*Daphnia rosea sensu* Uéno, Hoshino & Mizuno (1959)), our taxonomic sampling targeted the *Daphnia longispina* complex of the Palearctic. We also emphasized comparison of the specimens from Misumi-ike with Japanese populations of this complex (this includes two collections, Hourai-numa and Imori-ike, with putative new species from Ishida et al., 2011). Additional sequences were obtained from GenBank, including the only existing reference genome in the group (*Daphnia galeata* from Nickel et al., 2021). Zooplankton samples were collected and examined using standard methods (as in Garibian et al., 2021) with a net mesh size of 50 µm. Specimens of *Daphnia* were presorted with ethanol-preserved samples being selected under a binocular stereoscopic microscope Leica MZ7.5. They were then studied in toto under optical microscopes (Olympus CХ 41) in a drop of a glycerol-ethanol mixture. Then, 10 parthenogenetic females, five ephippial females, two juvenile and three adult males were dissected under a stereoscopic microscope using tungsten needles (Dumont & Negrea, 2002) to analyse appendages and postabdomens. Drawings were prepared using a camera lucida attached to the optical microscope. Morphological comparisons of the named species in the group in the far eastern Palearctic, and *Daphnia cucullata* were made with information in the literature (Table 2 and in the differential diagnoses). Taxonomic descriptions used standard anatomical terms for the genus *Daphnia* following Kotov et al. (2021).

**Table 2:** Comparison of the morphological characters for four members of the *Daphnia longispina* complex found in Japan and *Daphnia japonica* sp. nov.

Character	*D. galeata* (including *D. galeata mendotae*)	*D. cucullata*	*D. japonica* sp. nov.	*D. dentifera*	*D. longispina*
**Parthenogenetic female**					
Helmet and dorsal crest	Present in many populations	Present in most populations	Absent	Absent	Absent
Medial keel on posterior head margin	High	Absent	Absent	Low	Low
Length of stiff seta on inner-distal portion of limb II (“rigid seta”)/ soft seta	<1/2	<1/2	>1/2	<1/2	1/2 or less
Antennule position	Far from tip of rostrum	On tip of rostrum	Far from tip of rostrum	Far from tip of rostrum	Far from tip of rostrum
Antennular body	Fully reduced	Fully reduced	As a very low mound	As a very low mound	As a very low mound, or fully reduced
**Ephippium**					
Dorsal margin with rare, minute spinules	+	?	–	+	+
**Juvenile female and male**					
Neck teeth	Usually absent, a single tooth found in few populations, but they may belong to hybrids *i.e*., with “*hyalina*”	Absent	Multiple teeth, in juvenile males on a strong “pedestal”	Present in many populations, “pedestal” presence is variable	Present in many populations, “pedestal” presence is variable
**Adult male**					
Helmet	Usually present	Usually present	Absent	Absent in ponds	Absent in ponds
Rostrum	Developed	Developed	Smoothed	Different	Different
Antero-dorsal head extremity fully occupied by a huge eye vesicle	–	–	+	Different	Different
All three abdominal projections small	–	–	+	–	–
Antenna I long	–	–	+	Different	Different
Male length (extremes)	0.89–1.36	0.6–1 mm	1.3–1.6 mm	c.a. 0.9 mm	0.7–1.7 mm

**Note:**

We used these additional sources of information for *D. galeata*, *D. cucullata, D. dentifera* and *D. longispina* s. lat.: Brooks, 1957; Ueno, 1972; Mäemets, 1976; Flössner & Kraus, 1986; Alonso, 1996; Benzie, 2005; Hudec, 2010; Kirdyasheva & Kotov, 2013, 2019; Zuykova, Bochkarev & Katokhin, 2013; Zuykova et al., 2018a. Life stages for a given character are shown in bold font. Extreme body size measurements are given.

## Results

**Figure 1:**
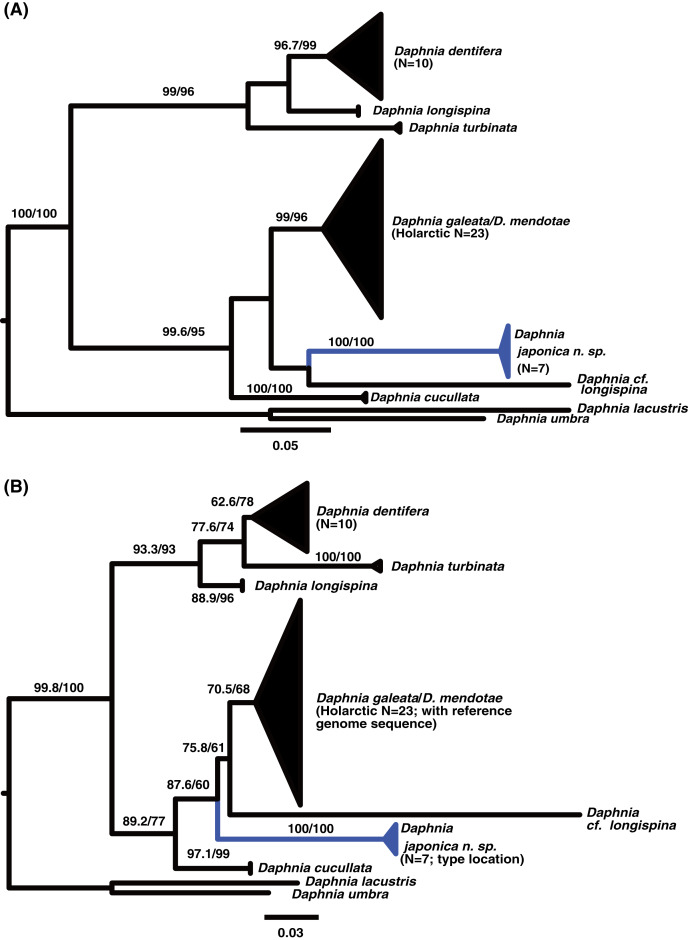
Maximum Likelihood phylograms of the *Daphnia longispina* complex based on the mitochondrial ND2 gene. Labeled triangles represent collapsed branches of *Daphnia galeata/mendotae*, *Daphnia dentifera*, and a putative new species *Daphnia japonica* sp. nov. (blue). Numbers above the branches indicate support values (approximate likelihood ratio tests/ultrafast bootstrap values). (A) Tree based on the nucleotide sequences of the ND2 coding region of the mitochondrial genome. (B) Tree based on the amino acid sequences of the ND2 coding region of the mitochondrial genome.

**Figure 2:**
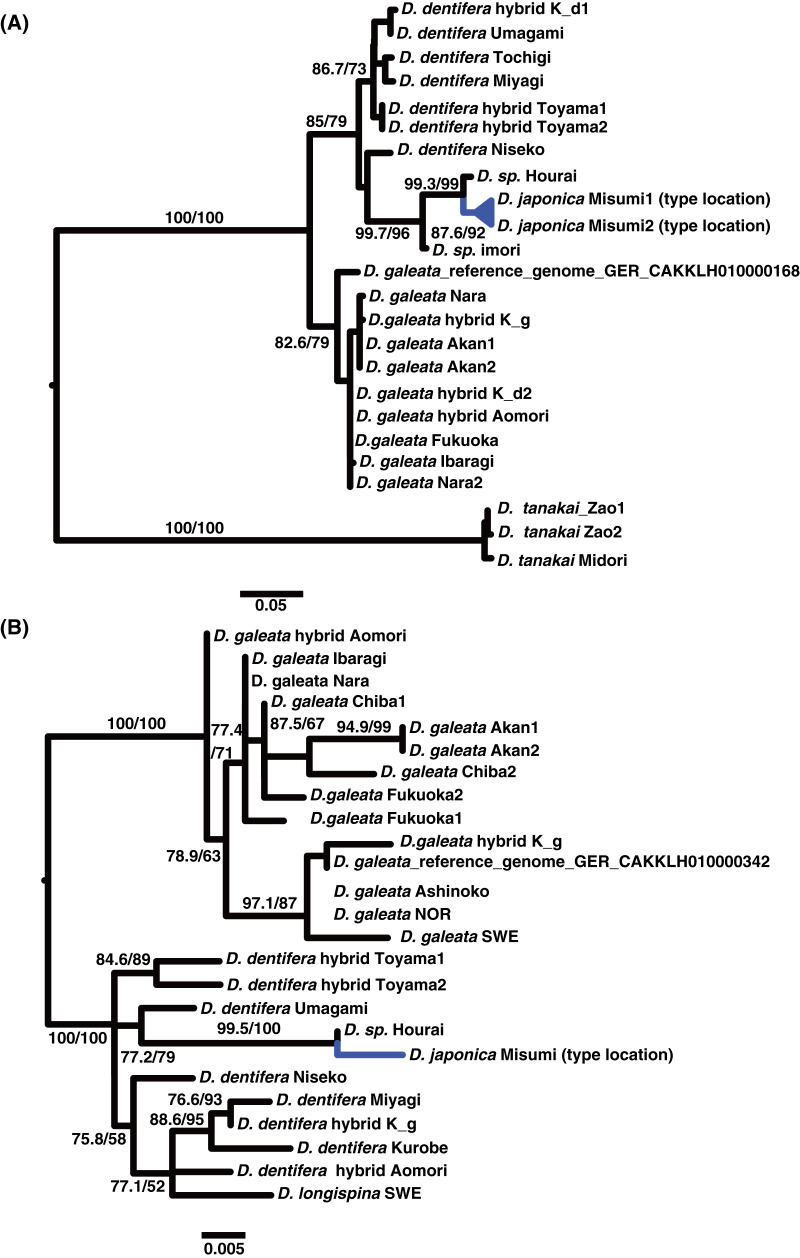
Maximum Likelihood phylograms of the *Daphnia longispina* complex based on nuclear non-coding loci one and two. Sequenced specimens are from the *Daphnia galeata mendotae*, *Daphnia dentifera*, and a putative new species *Daphnia japonica* sp. nov. (blue). Alleles from putative recent hybrids between *D. dentifera* and *D. galeata* are shown as hybrid. Numbers above the branches indicate support values (approximate likelihood ratio tests/ultrafast bootstrap values). (A) Tree based on non-coding nuclear locus 1. (B) Tree based on non-coding nuclear locus 2.

The alignment lengths were 930 nt for the ND2 gene, 480 nt for nuclear locus 1, and 501 nt for nuclear locus 2. The mitochondrial locus (Fig. 1) supported the genetic uniqueness and cohesiveness of the divergent lineages of *Daphnia: D. dentifera, D. longispina, D. turbinata, D. galeata, D. cucullata* and *D. japonica* sp. nov. Phylogenies of the nuclear loci supported the existence of *D. dentifera*, *D. galeata* and *D. japonica* sp. nov. as lineages (Fig. 2). However, for the nuclear loci, *D. dentifera* and *D. japonica* were not reciprocally monophyletic. No mitochondrial or nuclear sequence had a significant base composition difference from other sequences in their respective alignments (based on goodness of fit tests in IQtree). The test of relaxed selection for the mitochondrial branch leading to *D. japonica* was not significant (K = 0.17, *p* = 0.36). However, one published ND2 sequence (*Daphnia longispina*, JX069351) had the longest branch on the amino acid tree (Fig. 1B) and significant relaxed selection (K = 0.31, *p* = 0.001).

The nuclear genes showed monophyly of *D. japonica* sp. nov. with specimens from populations proposed by Ishida et al. (2011) as a new species. The ND2 sequences preserved the open reading frame. The phylogeny of ND2 revealed a placement of *D. japonica* sp. nov. within the *D. galeata/D.cucullata* clade using both nucleotide (Fig. 1A) and amino acid alignments (Fig. 1B). In contrast, nuclear locus 1 (Fig. 2A) and nuclear locus 2 (Fig. 2B) placed *D. japonica* sp. nov. within *D. dentifera*. However, the branches grouping *D. japonica* within *D. dentifera* were poorly supported, suggesting that the hypothesis of a sister group association between *D. japonica* and *D. dentifera* is also plausible. The average genetic distance of the novel lineage was 0.18 (K2P+G4) from *D. galeata* for ND2 and 0.12 (K2P+G4) from *D. dentifera* for nuclear locus one. For nuclear locus one, the divergent lineages from Honshu (*i.e*., *D. japonica* sp. nov. and the populations identified by Ishida et al., 2011) had an average within group genetic distance of 0.035.

**TAXONOMY**

**Order Anomopoda Sars, 1865**

**Family Daphniidae Straus, 1820**

**Genus *Daphnia* O.F. Müller, 1785**

**Subgenus *Daphnia* ( *Daphnia*) O.F. Müller, 1785**

***Daphnia* ( *Daphnia*) *longispina* species complex**

***Daphnia japonica* sp. nov.**

Figures 3–7

**Figure 3:**
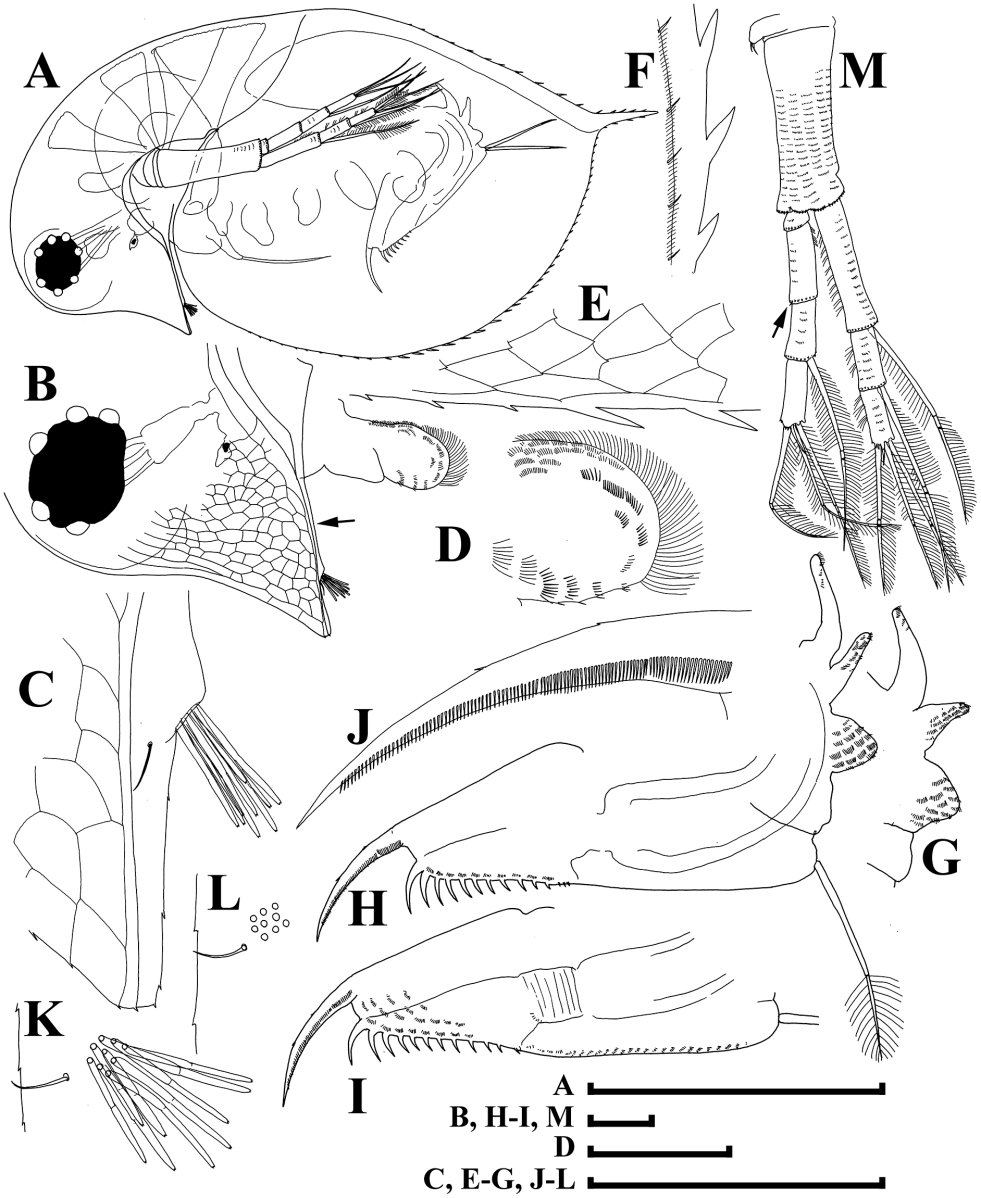
*Daphnia japonica* sp. nov., parthenogenetic female from Misumi-Ike, Yamagata Prefecture, Japan. (A) Lateral view. (B) Head, lateral view. (C) Rostrum. (D) Distal plate of labrum. (E) Postero-ventral portion of valve. (F) posterior valve margin, inner view. (G) Abdomen. (H and I) Postabdomen. (J) Postabdominal claw. (K) Antenna I, lateral view. (L) Antenna I, posterior view. (M) Antenna II. Scale bars: (A) = 1 mm; (B–M) = 0.1 mm.

**Figure 4:**
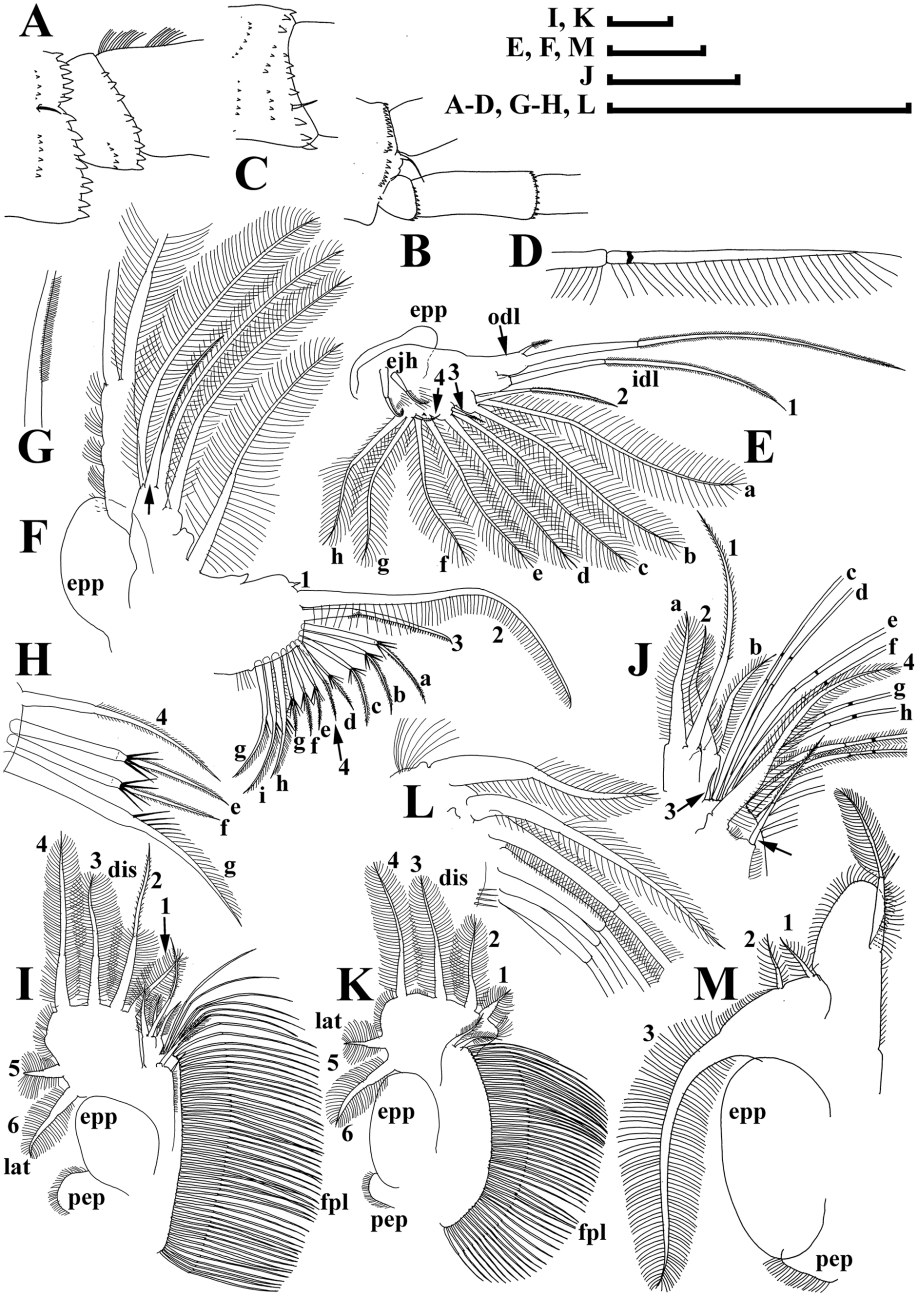
Illustrations of *Daphnia japonica* sp. nov., parthenogenetic female from Misumi-ike, Yamagata Prefecture, Japan. (A) Distal portion of antenna II basal segment, outer view. (B) Inner view. (C) Second exopod segment. (D) Distal swimming seta. (E) Limb I. (F) Limb II. (G) Outer seta on its inner-distal portion. (H) Setae of gnathobase. (I) Limb III. (J) Its inner-distal portion. (K) Limb IV. (L) its inner-distal portion. (M) Limb V. Scale bars: (I) K = 1 mm; (A–H, J, L and M) = 0.1 mm.

**Figure 5:**
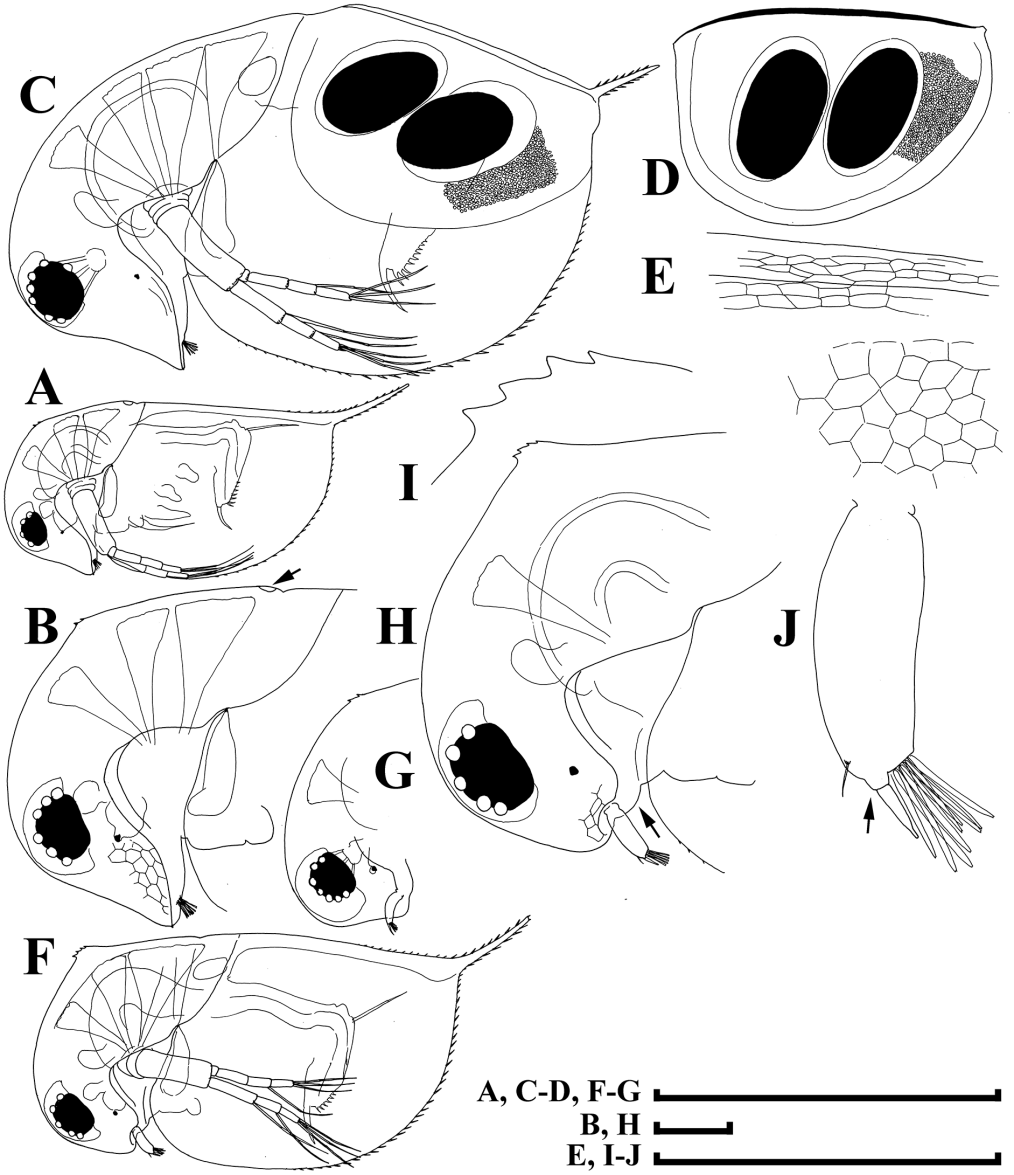
Illustrations of *Daphnia japonica* sp. nov. from Misumi-ike, Yamagata Prefecture, Japan. (A) Juvenile female, general view. (B) Head. (C) Ephippial female. (D) Ephippium. (E) Its dorsal portion. (F) Juvenile male. (G and H) Head. (I) Neckteeth. (J) Antenna I. Scale bars: (A, C, D, F, G) = 1 mm; (B, E, H–J) = 0.1 mm.

**Figure 6:**
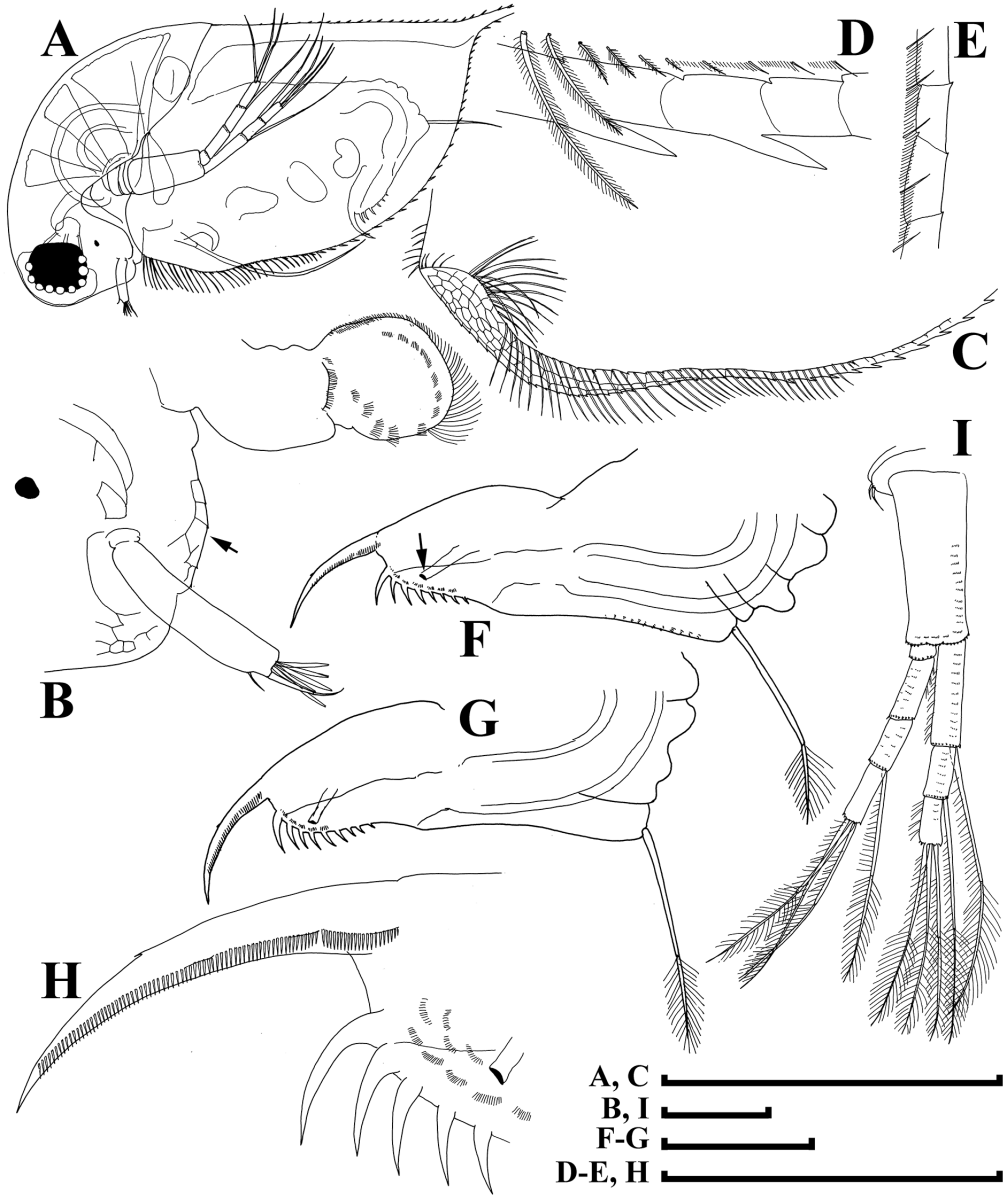
Illustrations of *Daphnia japonica* sp. nov., adult male from Misumi-ike, Yamagata Prefecture, Japan. (A) General view. (B) Head. (C) Ventral margin, inner view. (D) Postero-ventral valve portion. (E) Posterior margin, inner view. (F, G) Postabdomen. (H) Its distal portion. (I) Antenna II. Scale bars: (A, C) = 1 mm; (B, D–I) = 0.1 mm.

**Figure 7:**
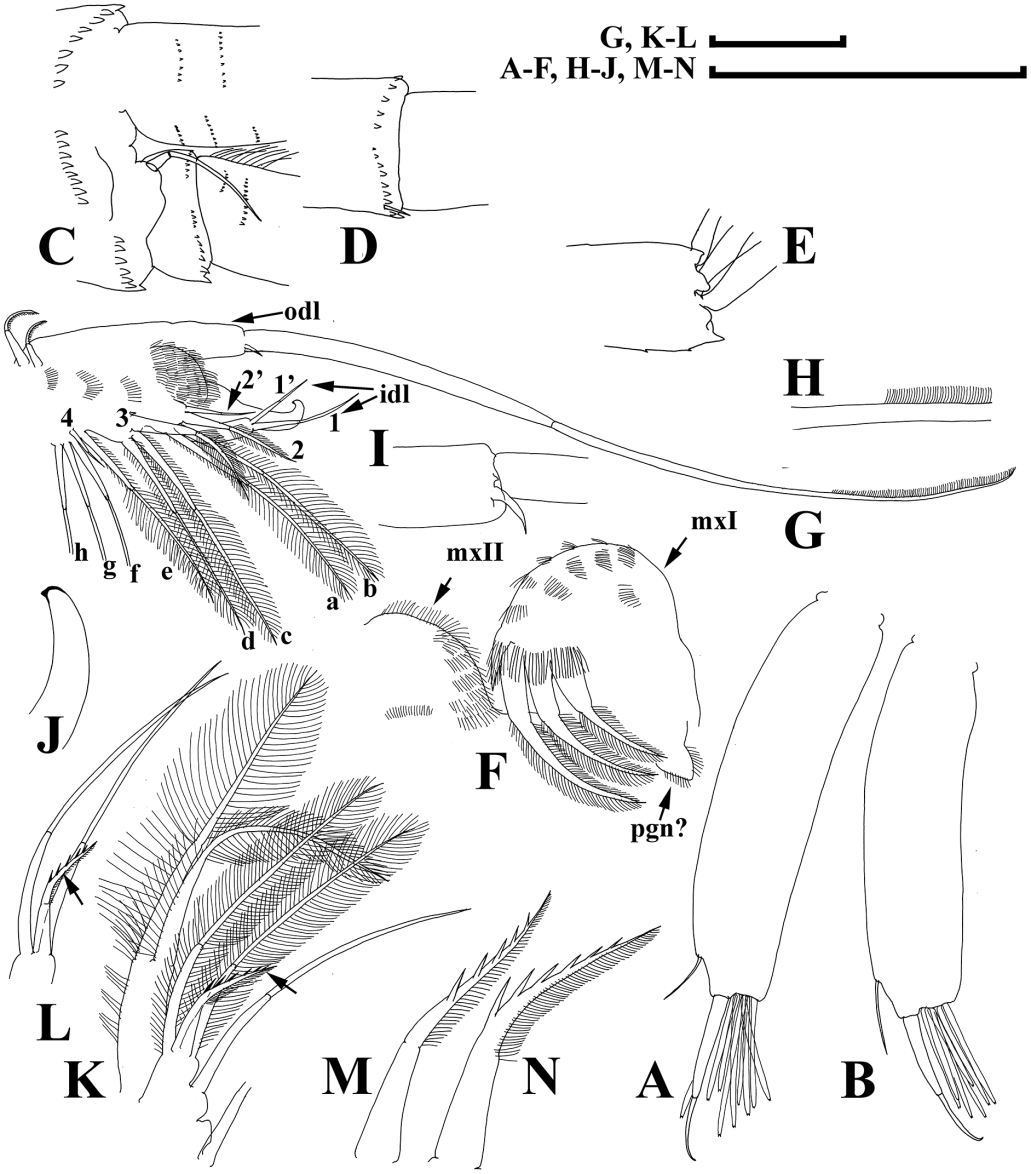
Illustrations of *Daphnia japonica* sp. nov., adult male from Misumi-ike, Yamagata Prefecture, Japan. (A and B) Antenna I. (C) Distal portion of antenna II basal segment, inner view. (D) Second segment of exopod. (E) Distal segment of exopod. (F) Paragnath, maxilla I and maxilla II. (G) Limb I. (H) Largest seta of its outer distal lobe. (I) Smallest seta of its outer distal lobe. (J) Tip of copulatory hook. (K and L) Inner-distal portion of limb I. (M and N) Stiff seta on it. Scale bars: (G, K and I) = 1 mm; (A–F, H–J, M and N) = 0.1 mm.

*Daphnia rosea* Sars in Uéno, Hoshino & Mizuno, 1959: 178–179, Figs. 3A–3D (Not *Daphnia rosea* Sars, 1862: 268–269).

“New species lineage” in Ishida et al., 2011: Fig. 2.

**Etymology.** The species is named after the country of the type locality—“of Japan” in Latin.

**Type locality.** A small, shallow bog-lake Misumi-Ike (38.3681,°N, 139.8210°E; about 1,060 m.a.s.l., max. depth 2.8 m, area ca. 1,700 m^2^), Yamagata Prefecture, N part of Honshu Island, Japan. The type series was collected in October 2006 by S. Ishida.

**Holotype.** A parthenogenetic female, MGU Ml-253 in the Collection of Zoological Museum of Moscow State University, Moscow, Russia.

**Allotype.** Adult male, MGU ML-254.

**Additional material examined from the type locality.** Fifty juvenile males and females, adult parthenogenetic, ephippial females and males, MGU Ml-255. Many juvenile males and females, adult parthenogenetic, ephippial females and males, AAK M-300 in the personal collection of A.A. Kotov, A.N. Severtsov Institute of Ecology and Evolution, Moscow, Russia.

**Short diagnosis.** Parthenogenetic female with very large head, low anterior crest (but without traces of helmet) and long, straight rostrum; posterior margin of head flat. Abdomen with first abdominal process almost straight, directed anteriorly, second process shorter, third process as a massive mound on the segment, fourth process absent. Antenna I body as minute hillock. Length of stiff seta on inner-distal portion of limb II (=rigid seta) 2/3 length of soft seta. Juvenile females and males with few neckteeth. Dorsal wall of ephippium forming dorsal plate without spinules. Adult male head with rounded rostrum, massive projection posterior to antenna I base. Distal most head extremity projected ventrally, fully occupied by large compound eye. Abdomen with all abdominal processes reduced, small mound present on each first, second and third segments. Gonopore opens subdistally, without genital papilla. Antenna I (antennule) long, almost straight; antennular setae small, its tip projected beyond antennular body tip. Male seta (flagellum) located on low process, longer than aesthetascs, bisegmented, with hooked tip. Length of females 0.9–2.1 mm, adult males 1.3–1.6 mm.

**Adult parthenogenetic female.** Body subovoid in lateral view, maximum height in body middle (Fig. 3A). Dorsal margin regularly convex, depression between head and rest of body absent. Postero-dorsal angle with very short caudal spine, ventral margin regularly convex. Head very large, with low anterior crest and long, straight rostrum, its tip subdividing into two lobes by a “line” of pre-rostral fold (Figs. 3B and 3C); posterior margin of head flat or slightly convex, without medial keel (Fig. 3B, arrow); ventral margin of head concave. Compound eye large, lying ventral to middle body axis and out of anterior most extremity of head; ocellus small. Labrum as rectangular main body with large, setulated distal labral plate (Figs. 3B, 3D). Carapace subovoid, spinules present on caudal spine and occupying more than a third of dorsal and a half of ventral valve margin (Figs. 3A, 3E). Inner face of posterior valve portion with fine setules, separated into groups by longer setules (Fig. 3F). Abdomen with first (basal most) abdominal process almost straight, directed anteriorly, second (middle) process shorter, third process as massive mound on the segment, fourth process absent (Figs. 3G and 3H). Postabdomen elongated, tapering distally, with ventral margin straight. Preanal margin long, concave; preanal angle smooth, postanal angle smooth. Paired spines on postanal angle and anal portion, their size increasing distally (Figs. 3H and 3I). Postabdominal seta as long as preanal margin, its distal segment shorter than basal armed by a continuous row of thin setules (Fig. 3H). Postabdominal claw with a continuous row of fine setules; pectens not distinct (Fig. 3J).

Antenna I body as minute hillock bearing nine aesthetascs, antennular seta arising immediately from surface of head (Figs. 3C, 3K and 3L); tips of aesthetascs do not reach tip of rostrum (Fig. 3B). Antenna II (Figs. 3A, 3M) with short coxal part possessing two very short sensory setae, basal segment elongated, a minute distal spine at its anterior face (Fig. 4A), a relatively long distal sensory seta on its posterior face (Fig. 4B). Antennal branches elongated, with four-segmented exopod and three-segmented endopod, small spine on distal end of second exopod segment (Fig. 3M, arrow; Fig. 4C). Antennal formula: setae 0-0-1-3/1-1-3. Each swimming seta with bilaterally setulated basal segment and unilaterally setulated distal segment, a chitinous band inserted within distal segment near joint with basal segment (Fig. 4D).

Limb I with an ovoid epipodite (Fig. 4E; epp), without accessory seta; outer distal lobe (Fig. 4E: odl) with long seta bilaterally armed distally with short setules, and short, thin, setulated seta; inner distal lobe (Fig. 4E: idl), or endite 4, with single, long anterior seta (Fig. 4E: 1); endite 3 with medium-sized seta (2), and two posterior setae (a–b); endite 2 with short anterior seta (3) and two posterior setae (c–d); endite 1 with small anterior seta (4), and four posterior setae (e–h). Two ejector hooks (ejh) of similar length.

Limb II with large, ovoid epipodite (Fig. 4F; epp); distal portion as large lobe bearing two long soft, setulated setae; four endites supplied by five setae, among them, stiff, anterior seta = rigid seta (Fig. 4F, arrow) about 1/2 to 2/3 of the length of other setae on distal most endite, unilaterally setulated distally (Fig. 4G); gnathobase with four anterior setae (Fig. 4F: 1–4) and 10–11 posterior setae of gnathobasic filter plate (a–g), seta 4 as long as e or f (Fig. 4H).

Limb III with globular pre-epipodite (Fig. 4I: pep), globular epipodite (epp) and flat exopodite bearing four distal (dis, 1–4) and two lateral (lat, 5–6) setae, seta 2 as long as 4, with short setules distally; inner-distal portion of limb with endite 4 bearing a single, long anterior seta (Fig. 4J: 1) and shorter posterior (a) seta; endite 3 with single anterior seta (2) and single posterior (b) seta of similar size; endite 2 with rudimentary anterior seta (3) and two posterior setae (c–d); endite 1 with large anterior seta, bilaterally armed by relatively long setules (4) and four posterior (e–h) setae. Gnathobase bears numerous filtering setae (Fig. 4I: fpl), and single, relatively long anterior seta armed by short setules (Fig. 4J: arrow) in its distal corner.

Limb IV (Fig. 4K) with small pre-epipodite (pep), large, ovoid epipodite (epp) and exopodite bearing four distal (1–4) and two lateral (5–6) setae. Inner-distal portion of this limb with completely fused endites, distally with two setae of unclear homology (Fig. 5L), much of limb inner margin is a gnathobase filter plate consisting of numerous filtering setae (fpl).

Limb V (Fig. 4M) with small, setulated pre-epipodite (pep), large, subovoid epipodite, triangular exopodite supplied with two short distal setae (Fig. 4M: 1–2), large lateral seta (3); inner limb portion as ovoid flat lobe, with setulated inner margin and single, large seta.

**Juvenile female.** Body more elongated, with straight posterior margin and longer caudal spine (Fig. 5A). Head with straight ventral margin, rostrum short, dorsal margin convex, with 1–3 neckteeth, dorsal organ in posterior portion of head (Fig. 5B: arrow), but anterior to small notch or depression.

**Ephippial female.** A depression between head and valves, dorsal margin of valves straight (Fig. 5C). Ephippium with two resting eggs, with axes being almost perpendicular to ephippial dorsal margin, egg chambers not separated from each other, much of ephippium darkly pigmented and covered with sculpturing of polygonal cells, postero-dorsal portion of valves with caudal spine incorporated into ephippium (Figs. 5C, 5D). Dorsal wall of carapace forming dorsal plate without spinules (Fig. 5E).

**Juvenile male of pre-reproductive instar.** Body elongated, dorsal margin straight, rudimentary depression between head and valves, postero-dorsal angle with short caudal spine (Fig. 5F). Head with rounded rostrum (Figs. 5G and 5H), strong rounded depression dorsal to antenna I base (Fig. 5H: arrow), ventral margin straight, anterior-most extremity of head not projected; 4–5 neckteeth located on special projection (“pedestal”) (Fig. 5I). Eye medium-sized, ocellus small. Valve with antero-ventral angle not prominent, small denticles at ventral margin, but no setae. Antenna I short, with fine antennular sensory and short male seta (shorter than aesthetascs) located on top of low distal process (Fig. 5J: arrow).

**Adult male.** Body elongated, dorsal margin of valves straight, depression between head and valves shallow, postero-dorsal angle with long caudal spine (Fig. 6A). Head with rounded rostrum, massive projection posterior to antenna I base (Fig. 6B: arrow). Distal-most head extremity projected ventrally, fully occupied by large compound eye, ventral head margin slightly concave. Valve with antero-ventral angle distinctly prominent ventrally, entire ventral margin with numerous setae, located submarginally (on inner face of valve) in anterior and posterior portions of valve (Figs. 6C and 6D). A row of setules on inner face of posterior margin subdivided into groups by longer setules (Fig. 6E). Abdomen with all abdominal processes reduced, only small mound present on each first (basal most), second and third segments (Figs. 6F and 6G). Postabdomen with slightly convex ventral margin; preanal margin concave, preanal angle expressed, postanal angle rounded. Gonopore (Fig. 6F: arrow) opens subdistally, without genital papilla. On outer surface of postabdominal claws, three pectens composed of thin setules with a modest separation of proximal pectens (Fig. 6H). Antenna I long, almost straight; antennular setae small, its tip projected beyond antennular body tip. Male seta (flagellum) located on low process, longer than aesthetascs, bisegmented, with hooked tip (Figs. 7A and 7B). Antenna II as in female (Figs. 6I, 7D and 7E), but sensory seta on inner side of basal segment in its distal portion long (Fig. 7C). Maxilla II (rarely studied in *Daphnia*) present in male (Fig. 7F: mxII) together with maxilla I and paragnath (pgn).

Limb I without accessory seta, outer distal lobe large, cylindrical (Fig. 7G: odl), bearing very large seta supplied with minute setules distally (Fig. 7H) and a rudimentary seta (Fig. 7I); inner distal lobe (Fig. 7G: idl) with bent copulatory hook, and two setae of different size (Fig. 7G: 1 and 1′); endite 3 with 4 setae (additional seta marked as 2′), both setae 2 and 2′ relatively short, seta 3 remarkably larger than in female, seta 4 somewhat larger than in female. Limb II: distal most endite with a short, hook-like anterior seta (Figs. 7K and 7L: arrow), with setulated distal segment, along one side (Figs. 7M and 7N).

**Size.** Size of holotype: 1.77 mm, parthenogenetic females 0.9–2.1 mm (*n* = 20), ephippial females 1.7–2.0 mm (*n* = 5), juvenile males 0.7–0.8 mm (*n* = 10), adult males 1.3–1.6 mm (*n* = 5).

**Differential diagnosis.** *D. japonica* sp. nov. differs from the species of the *D. longispina* species complex known from Japan in a series of characters (See Table 2). *D. japonica* can be differentiated from *D. galeata* by the absence of the helmet and a straight or slightly curved posterior head margin lacking a medial keel in females and a very massive antero-ventral head extremity fully occupied by a large compound eye. Unlike the males of *D. japonica*, the males of the lacustrine *D. ezoensis* Ueno, 1972 possess a rostrum and lack pronounced ventral anterior margin setation. The females of *D. ezoensis* have a lower head shape (see Tanaka, 1997) as compared to *D. japonica* sp. nov. *Daphnia dentifera* is most easily confused with *D. japonica*. The females of *D. japonica* sp. nov. have a longer rigid seta on limb II as compared to females of *D. dentifera*, and the medial keel is fully absent in *D. japonica* sp. nov. but is present in *D. dentifera*. In contrast to *D. cucullata*, aesthetascs in *D. japonica* sp. nov. are protruding on a small mound located far from the rostrum tip. In contrast to *D. longispina*, the ephippium of *D. japonica* sp. nov. lacks spinules on the posterior margin. Finally, the male abdominal processes is are small and indistinct in *D.japonica sp. nov*., which is not the case in other species considered here.

**Distribution.** *D. japonica* was likely studied and illustrated from Ôtori-Misumi-Ike as *D. rosea* by Uéno, Hoshino & Mizuno (1959). However, we are uncertain of the distribution of *D. japonica* beyond the type location of Misumi-Ike. We found nearly identical nuclear sequences to those of the type location in Hourai-numa Pond, Iwate (40.6096°N 140.9390°E, 685 m.a.s.l.) and Lake Imori-ike (in reality, this is a pond c.a. 20 × 50 m), Niigata (36.6314°N, 138.5361°E, 1,335 m.a.s.l.) (populations also studied by Ishida et al., 2011). We tentatively propose that *D. japonica* is distributed in mountain ponds and bog-lakes, on Honshu Island, Japan.

## Discussion

### Evidence of a new species

The genetic and morphological evidence supports the existence of a novel species of *Daphnia* present on Honshu, Japan. The strong support for monophyly and pronounced divergence at two nuclear loci and the mtDNA locus is consistent with the species hypothesis. The nDNA supports a grouping of the Misumi-Ike population with specimens from Imori-ike and Hourai-numa populations that contained a putative new species proposed by Ishida et al. (2011). mtDNA sequences of specimens from all three populations are also divergent from known species (Misimu-ike from this study and Imori-ike, Hourai-numa from Ishida et al., 2011; Beninde, 2021). Although we used the same collections as Ishida et al. (2011) for Hourai and Imori, assignment of the earlier mitochondrial lineages (Hourai-numa, Imori-ike) to *D. japonica* awaits direct comparison with the mitochondrial markers used by Ishida et al. (2011). The open reading frames of ND2 sequences from Misumi-Ike and the lack of significant relaxed selection are inconsistent with the mitochondrial pseudogene hypothesis. Also, the strong divergences (18% for mtDNA) between this lineage and known species make incomplete lineage sorting less likely as an explanation for the novelty of this group. As we included samples from other Holarctic regions in the mtDNA analysis, it is unlikely that the divergences represent cryptic invaders. We failed to detect other lineages coexisting with *D. japonica* sp. nov., but increased sampling may change this finding. The discordance in the association of mtDNA and noncoding nDNA/morphology is consistent with ancient asymmetric introgression (and perhaps capture of a divergent mtDNA lineage). Beninde (2021) found a similar discordance for this lineage (not including samples from Misumi-Ike) using genome-scale data. It is unknown if the putative mitochondrial donor species is extant.

As the *Daphnia longispina* complex is a geographically widespread syngameon (Taylor, Sprenger & Ishida, 2005), introgression has been invoked for polymorphic populations with discordant mtDNA haplotypes (Thielsch et al., 2017; Schwenk, Ender & Streit, 1995). Recent stabilized introgressants have been proposed for the formation of *D. mendotae* (Taylor & Hebert, 1993; Taylor, Sprenger & Ishida, 2005) and some members of the *D. pulex* group (Marková et al., 2013). Ishida et al. (2011) predicted the existence of stabilized ancient (*i.e*., pre-glacial) introgressed lineages in weakly glaciated regions such as Japan. We have no calibrated molecular clock for among-population divergences that includes Misumi-ike specimens. If the lineages of Ishida et al. (2011) do belong to *D. japonica*, then the divergences for mtDNA among populations suggest that the species origin predates the last glaciation. Indeed, Beninde (2021) estimated that this lineage shared a common ancestor with *D. dentifera*/*D. longispina* about 15 MYA.

There is increasing support from the genomic studies that stabilized members of syngameons can and should be taxonomically recognized despite a lack of complete reproductive isolation (Cannon, 2021). Presently, it is unknown if *D. japonica* sp. nov. can coexist with extant members of the complex. However, members of the *D. longispina* complex are almost never detected in very low pH waters (Iwabuchi et al., 2017). Indeed, one of the most acid-tolerant species, *D. dentifera*, fails to survive and grow at a pH of 4.5. In contrast, *D. japonica* sp. nov. has been detected in Misumi-Ike when the pH was measured as low as 4.4 (Uéno, Hoshino & Mizuno, 1959). Given the genomic, morphological and ecological cohesion of the divergent lineages that we observed, we propose a new species *Daphnia japonica* sp. nov.

### Morphological evolution and phenotypic plasticity

In addition to genetic markers, we were able to identify several taxonomically informative morphological traits. Several of these traits (such as head shape) are usually phenotypically plastic, but plasticity need not preclude taxonomic informativeness. For example, the reaction norms of helmet shape for the *longispina* complex in Japan are nonoverlapping between *Daphnia galeata* and *Daphnia dentifera* (Ishida et al., 2011). Likewise, fully developed inducible neckteeth appear to be absent from *D. galeata*. Juveniles in a few populations bear a single tooth on the head (Glagolev, 1986), but such populations, when examined genetically, have been assigned to early generation hybrids (Taylor & Hebert, 1992). We have also found that the rigid seta length ratios are informative in East Asia for this species complex. Although this seta length ratio is thermally plastic in *Daphnia galeata* (Kim & Taylor, 2022), *D. japonica* has a larger warm-water ratio (>10 °C) than is known for *D. galeata*. Non-plastic morphological differences among species of the *Daphnia longispina* complex (especially among the parthenogenetic females) are few in number. However, the abdominal processes of parthenogenetic females and males appear to have diagnostic value in the group (Brooks, 1957; Ueno, 1972).

As the mitochondrial gene trees group *D. japonica* sp. nov. as a divergent lineage related to *D. galeata*/*D. cucullata* (*i.e*., discordant with morphology), there may be an undetected species in this group that donated this genome to *D. japonica* sp. nov. The results support the contention that the analyses of both nuclear and mitochondrial markers are necessary when divergent mitochondrial haplotypes are detected in this species complex (Thielsch et al., 2017). Presently, *D. japonica* sp. nov. is an endemic taxon known only from Honshu.

Our findings follow a series of discoveries of montane and subarctic lineages of the *Daphnia longispina* complex (Taylor & Hebert, 1994; Taylor, Hebert & Colbourne, 1996; Nilssen et al., 2007; Zuykova et al., 2018b). Populations from such regions with a *longispina*-like morphology must be carefully assessed for cryptic species. Some of these are likely undescribed species, such as the *Daphnia* from Berse in Norway (Petrusek et al., 2008; Zuykova, Bochkarev & Katokhin, 2013; Zuykova et al., 2018a). Understanding hidden diversity in these relict habitats warrants research priority as these waters are very sensitive to anthropogenic changes such as eutrophication, climate warming, and species introductions.

### The Eastern Palearctic as a center of cladoceran endemism

Here we describe a new species of *Daphnia* that is likely present in but a few mountain lakes and ponds in Japan. The daphniid fauna of many Japanese and other eastern Palearctic waters have recently been studied in detail with genetic markers (Ishida, Kotov & Taylor, 2008; Ishida et al., 2011; Ishida & Taylor, 2007a; So et al., 2015). Also noteworthy is that well-studied temperate species have populations in East Asia that are genetically divergent from western Palearctic populations (Ishida & Taylor, 2007a; Ishida & Taylor, 2007b; Ma et al., 2014; Tokishita et al., 2017). Palearctic montane and subarctic specialists such as *Daphnia umbra* and *Daphnia lacustris* are presently unknown from the eastern Palearctic (Zuykova et al., 2018b).

Korovchinsky (2006) proposed that the subtropics and temperate regions were centers of ancient (Tertiary) cladoceran endemism. Indeed faunistic, taxonomic and molecular studies have revealed numerous endemics in East Asia: the Russian Far East, China, Korea and Japan (Ishida, Kotov & Taylor, 2008; Kotov et al., 2021; Kotov et al., 2011a; Kotov et al., 2011b; Ma et al., 2019; Makino et al., 2020; Maruoka et al., 2018; Smirnov & Sheveleva, 2010; Yamamoto, Makino & Urabe, 2020). Direct phylogeographic evidence of a pre-Pleistocene age of some phylogeographic patterns have also been obtained (Kotov et al., 2021). Other taxa in this region are apparently of southern origin as they are thermophiles with a pronounced northern range limit (Garibian, 2017; Kotov, 2016; Kotov, Jeong & Lee, 2012; Kotov et al., 2011a; Kotov et al., 2011b). Regarding the Holarctic pattern, it is expected that East Asia would have suffered less extinction of temperate endemics compared to other regions (such as the Nearctic) because of the reduced influence of Pleistocene glaciations. *D. japonica* sp. nov. appears to represent such a pre-Pleistocene relict in agreement with Korovchinsky (2006).

## Conclusions

We find genetic and morphological evidence that supports the existence of a new endemic species of *Daphnia* from East Asia. We describe *Daphnia japonica* sp. nov. from a montane bog on Honshu Island. The mitochondrial phylogeny was discordant with the morphological and nDNA similarity to *D. dentifera*. The finding continues a trend of hidden diversity in ecologically sensitive montane habitats and in East Asia for *Daphnia*.

## Supplemental Information

10.7717/peerj.14113/supp-1 Supplemental Information 1 Accession numbers for mitochondrial ND2 sequences used in the present study from databases for the *Daphnia longispina* complex. Locations for the NCBI sequences of the ND2 mitochondrial gene region used in the present study.

10.7717/peerj.14113/supp-2 Supplemental Information 2 DNA sequences and alignments from Daphnia japonica. Anonymous nuclear local alignments and DNA sequences from mitochondrial ND2 for Daphnia japonica in FASTA format.

## Abbreviations in illustrations and text

dis: distal setae of exopodite
ejh: ejector hooks on limb I
epp: epipodite
ext: exopodite
fpl: filter plate of gnathobase
lat: lateral setae of exopodite
odl: outer distal lobe of limb I
pep: preepipodite

## Additional Information and Declarations

### Data Availability

The following information was supplied regarding data availability: The DNA sequences of Daphnia are available at GenBank: OL412563–OL412665 and OL362043–OL362049.

### New Species Registration

The following information was supplied regarding the registration of a newly described species: Publication LSID: urn:lsid:zoobank.org:pub:7AAA1961-81B8-46D8-8D0A-9FE1F5CD776D. *Daphnia japonica* Kotov & Taylor name: urn:lsid:zoobank.org:act:CCA5F28A-DB95-40B6-A1A4-E92099C8611C.
