# Supplementary material for: Daphnia japonica sp. nov. (Crustacea: Cladocera) an eastern Palearctic montane species with mitochondrial discordance
Source: PeerJ. 2022 Oct 4;10:e14113. doi: 10.7717/peerj.14113 (PMC9541614; doi:10.7717/peerj.14113)
Supplement: Supplemental Information 1 — Locations for the NCBI sequences of the ND2 mitochondrial gene region used in the present study. [file peerj-10-14113-s001.docx]

Table S1. **Locations and Genbank Accession numbers for mitochondrial sequences of the *Daphnia* *longispina* complex (including *Daphnia japonica* sp. nov. from this study).**

|  | Country | Site | Latitude | Longitude | NCBI Accession |
| --- | --- | --- | --- | --- | --- |
| *Daphnia cucullata* | Germany | Storkowersee | 52˚14'N | 13˚58'E | [DQ980402](https://www.ncbi.nlm.nih.gov/nuccore/DQ980402.1) |
| *D. cucullata* | Poland | Dargin Lake | 54˚8'N | 21˚44'E | [DQ980403](https://www.ncbi.nlm.nih.gov/nuccore/DQ980403.1) |
| *D. dentifera* | British Columbia, Canada | Moose Lake | 52˚59'N | 119˚0'W | [DQ980404](https://www.ncbi.nlm.nih.gov/nuccore/DQ980404.1) |
| *D. dentifera* | California, USA | Sequoia Lake | 36˚44'N | 118˚59'W | DQ980405 |
| *D. dentifera* | Colorado, USA | Copeland Lake | 40˚13'N | 105˚32'W | - [DQ980406](https://www.ncbi.nlm.nih.gov/nuccore/DQ980406.1) |
| *D. dentifera* | Colorado, USA | Sprague Lake | 40˚19'N | 105˚36'W | - [DQ980407](https://www.ncbi.nlm.nih.gov/nuccore/DQ980406.1) |
| *D. dentifera* | Colorado, USA | Red Rock Lake | 40˚5'N | 105˚32'W | DQ980408 |
| *D. dentifera* | Indiana, USA | Crane Lake | 41˚17'N | 85˚29'W | DQ132624 |
| *D. dentifera* | Nepal |  |  |  | JX446620 |
| *D. dentifera* | New York, USA | Blue Pond | 43˚2'N | 77˚49'W | DQ980410 |
| *D. dentifera* | New York, USA | Crossman's Lake | 43˚2'N | 77˚28'W | DQ845271 |
| *D. dentifera* | Washington, USA | Crescent Lake | 48˚4'N | 123˚47'W | [DQ980411](https://www.ncbi.nlm.nih.gov/nuccore/DQ980411.1) |
| *D. dentifera* | Washington, USA | Quinault Lake | 47˚28'N | 123˚53'W | [DQ980412](https://www.ncbi.nlm.nih.gov/nuccore/DQ980411.1) |
| *D. dentifera* | Washington, USA | Shannon Lake | 48˚33'N | 121˚44'W | [DQ980413](https://www.ncbi.nlm.nih.gov/nuccore/DQ980411.1) |
| *D. galeata* | Hokkaido, Japan | Shikotsu Ko | 42˚46'N | 141˚24'E | DQ980355 |
| *D. galeata* | Honshu, Japan | Kawaguchi Ko | 35˚30'N | 138˚46'E | DQ980401, DQ980354 |
| *D. galeata* | Honshu, Japan | Ebisu Dam | 35˚7'N | 136˚16'E | DQ980353 |
| *D. galeata* | Honshu, Japan | Biwa Ko | 35˚15'N | 136˚5'E | DQ980352 |
| *D. galeata* | Honshu, Japan | Kizaki Ko | 36˚34'N | 137˚50'E | LC177070 |
| *D. galeata* | Honshu, Japan | Chuzenji Ko | 36˚44'N | 139˚28'E | DQ980280,  DQ980350 |
| *D. galeata* | Kyushu, Japan | Ikeda Ko | 31˚14'N | 130˚34'E | DQ980399 |
| *D. galeata* (**reference genome**) | Germany | Müggelsee | 52°26′N | 13°39′E | GCA_918697745.1 |
| *D. japonica* n. sp. | Japan | Misumi-ike | 38°22' N | 139° 49'E | OL362043-OL362049 (this study) |
| *D. longispina* | Finland | A pond near Muonio | 67˚36'N | 23˚33'E | DQ132610 |
| *D. longispina* | Russia | Chany Lake |  |  | KP253098 |
| *D. longispina* | Russia | Dodot Lake |  |  | KP253089 |
| *D. longispina sp.* | Czech Republic | Zelivka Reservoir |  |  | JX069351 |
| *D. mendotae* | Indiana, US | Center Lake | 41˚14'N | 85˚51'W | DQ980251 |
| *D. mendotae* | Ontario, Canada | Galeairy Lake | 45˚30'N | 78˚17'W | DQ132605 |
| *D. turbinata* | Mongolia | Khargal Nuur Lake | 49° 55' 28.2'' N | 102° 44' 56.76'' E | MK930505 |
| *D. turbinata* | Mongolia | Khargal Nuur Lake | 49° 55' 28.2'' N | 102° 44' 56.76'' E | MK930504 |
| *D. umbra* | Nunavut, Canada | Pond near Richards Bay, |  |  | DQ132609 |
